# Development and Validation of the Professional Readiness in Diversity and Equity Scale: Protocol for a Scale Validation Study

**DOI:** 10.2196/95456

**Published:** 2026-09-21

**Authors:** Nigel Sherriff, Massimo Mirandola, Laetitia Zeeman, Francesco Amaddeo, Ann C Zumwalt, Sharon M Coleman, Nina Cesare, Carl Streed Jr, Jörg Huber, Mandi Pratt-Chapman, Dustin Z Nowaskie, Markus Paul Bidell

**Affiliations:** 1 Centre for Transforming Sexuality & Gender, School of Education, Sport, and Health Sciences, University of Brighton, Brighton, England, United Kingdom; 2 Department of Diagnostics and Public Health, University of Verona, Piazzale Aristide Stefani 1, Epidemiology Unit - Division of Infectious Diseases, Verona, Verona, 37126, Italy, 39 045 8121111; 3 Department of Neurosciences, Biomedicine and Movement Sciences, University of Verona, Verona, Verona, Italy; 4 Department of Anatomy & Neurobiology, Boston University Chobanian & Avedisian School of Medicine, Boston, MA, United States; 5 Biostatistics and Epidemiology Data Analytics, Center for Health Data Science, Boston University School of Public Health, Boston, MA, United States; 6 Section of General Internal Medicine, Department of Medicine, Boston University Chobanian & Avedisian School of Medicine, Boston, MA, United States; 7 Cancer Center, George Washington University, Washington, DC, United States; 8 Keck School of Medicine, University of Southern California, Los Angeles, CA, United States; 9 Hunter College, City University of New York, New York, NY, United States

**Keywords:** lesbian, gay, bisexual, transgender, queer, and intersex, LGBTQI+, scale validation, public health, health professionals, cultural competence

## Abstract

**Background:**

Lesbian, gay, bisexual, transgender, queer, and intersex (LGBTQI+) patients continue to experience stigma, discrimination, and inadequate knowledge among mental health and medical professionals. These barriers can negatively impact health care outcomes, leading to delayed treatment, mistrust, and unmet medical needs within LGBTQI+ communities. Providing culturally competent care to LGBTQI+ people remains a critical challenge within health and social care. The development and use of reliable and valid assessments examining LGBTQI+ health care professional competency continue to be important in understanding and ameliorating LGBTQI+ health inequalities.

**Objective:**

The aim of the study is to develop and psychometrically validate a new multinational instrument designed to assess health care professionals’ LGBTQI+ attitudes, awareness, general skills, and knowledge, as well as to examine LGBTQI+ workplace affirmative policies, support, and climate.

**Methods:**

An initial pool of 100 new scale items was developed by reviewing existing LGBTQI+ health care scales and consulting with LGBTQI+ health care experts in the summer of 2023. Leading experts in LGBTQI+ health care were then asked to participate in the Delphi method to provide numeric and qualitative feedback on the 100 items. This process yielded a final primary survey comprising 39 test items. A cross-sectional multicenter, multicountry scale validation study using a web-based survey was administered to health care professionals and trainees in Italy, the United States, and the United Kingdom between October 2024 and December 2025 using a convenience sampling approach complemented by snowball recruitment. Research participants were asked to complete a demographic and professional survey, the 39 new test items, the 18-item Lesbian, Gay, Bisexual, and Transgender Development of Clinical Skills Scale (LGBT-DOCSS), and the 12-item Workplace Support subscale of the LGBT Climate Inventory (LGBTCI).

**Results:**

Funding commenced in May 2023, and data collection was conducted from October 2024 to December 2025. A total of 1339 eligible people completed the survey. Data analysis has started, and results will be reported in theautumn of 2026. It is anticipated that the Professional Readiness in Diversity and Equity (PRIDE) scale will emerge as a reliable and valid tool for assessing health care professionals’ LGBTQI+ attitudes, awareness, general skills, and knowledge, as well as examining LGBTQI+ workplace affirmative policies, support, and climate. The instrument may also serve as a useful outcome measure in research evaluating professional practice and development.

**Conclusions:**

The development and validation of the new PRIDE scale represent a significant advancement toward improved cultural competence among health care professionals. Validating the scale in both English- and Italian-speaking populations will support the development of international interventions and, ultimately, treatment outcomes for LGBTQI+ people. The PRIDE scale has the potential to exert a far-reaching impact on the delivery of clinical care and public health services worldwide.

## Introduction

A growing number of adults now identify as lesbian, gay, bisexual, transgender, queer, and intersex (LGBTQI+), even as this increase coincides with a rapid and broadening backlash against LGBTQI+ communities [1,2]. Health and social care systems remain critically challenged in providing adequate care to these populations [3-7]. LGBTQI+ people report higher rates of stigma and discrimination, as well as inadequate health care professional knowledge during health care encounters, compared with heterosexual and cisgender peers, which in turn leads to mistrust, delayed treatment, and unmet medical needs in the LGBTQI+ community [8-11]. These patterns are observed in multiple nations and ultimately lead to extensive health inequities in these populations worldwide [1,5].

It is incumbent upon the health care system to address the drivers of these systemic inequities [12,13]. One such driver is the inconsistent and often inadequate care provided by individuals within the health care system [6,7,14,15]. Health care professionals should consistently provide competent and inclusive care to LGBTQI+ individuals. Such care is grounded in respect for LGBTQI+ identity, acknowledges the unique life experiences of LGBTQI+ individuals, and is aware of the population-level health inequities experienced by these communities [16-18]. However, continuing negative experiences of LGBTQI+ people in the health care system emphasize that many health care professionals fail to provide this standard of care.

The principles of quality assurance in health care dictate that improving the health care system requires being able to systematically monitor, evaluate, and elevate standards of care [19,20]. To address the well-documented health inequities experienced by LGBTQI+ patients, it is therefore essential to be able to measure and address health care professionals’ knowledge, clinical skills, and prejudice toward the LGBTQI+ community [21]. Numerous psychometric tools have been developed to evaluate health care professionals’ clinical preparedness and attitudes toward LGBTQI+ patients. These tools play an essential role in identifying gaps in knowledge, biases, and areas for improvement in delivering inclusive and competent care. For example, the Lesbian, Gay, Bisexual, and Transgender Development of Clinical Skills Scale (LGBT-DOCSS) [22] evaluates key areas of competence in health and social care, including clinical preparedness, attitudinal awareness, and knowledge. The QUeering Individual Relational and Knowledge Scales (QUIRKS) compare health care professional and patient experiences in the health care system [23], and the Lesbian, Gay, Bisexual, and Transgendered Climate Inventory (LGBTCI) assesses the perceived inclusiveness of health care environments [24].

Of the scales that specifically focus on health care professionals, the LGBT-DOCSS is the most widely used across a variety of health care contexts [21]. However, this and many other instruments no longer fully reflect contemporary language, concepts, or understandings of LGBTQI+ identities. Over time, the terminology and concepts used to describe sexual orientation, gender identity, and gender expression have changed significantly, and these changes need to be reflected in new LGBTQI+ assessment scales. Outdated scales can be stigmatizing for LGBTQI+ people as well as contribute to perpetuating inequalities through the generation of poor quality and reduced construct validity. In addition, there is now greater awareness that organizational climate and support for LGBTQI+ people play an important role in driving health care professionals’ attitudes and behaviors toward this population [25-27]. Considering the impact of the LGBT-DOCSS, we acknowledge the need to develop a new LGBTQI+ cultural competency scale that is multinational in its development and psychometric evaluation, reflects more contemporary language and conceptual nuance, and incorporates assessment of workplace climate.

The overarching aim of this study is to develop and validate a new multinational psychometric instrument that can assess health care professionals’ LGBTQI+ attitudes, awareness, general skills, and knowledge, as well as examine perceptions of LGBTQI+ workplace climate. Development of the new Professional Readiness in Diversity and Equity (PRIDE) scale addresses the continual evolution of language and cultural understanding surrounding LGBTQI+ identities and the emergence of new challenges faced by LGBTQI+ people within health and/or social care settings. The objectives of this protocol are to outline the methodological framework used to design and validate the PRIDE scale. We begin by discussing how we generated our sample and how we designed candidate items and measures. We describe in detail the analytic methods used to identify and validate the factor structure and to evaluate the psychometric properties of the scale. Finally, we address plans for validating the applicability of this scale across demographic groups and international or institutional contexts. The resulting instrument is intended to provide a robust, multidimensional assessment of clinical competence among health care professionals in diverse cultural and organizational settings.

## Methods

### Scale Development and Measures

The PRIDE Scale included 39 new test items examining LGBTQI+ health care knowledge, awareness, attitudes, skills, and the work environment,. Development of these items was iterative and generated through literature reviews, expert consultation, and adaptation from existing scales. The Delphi method was used to select the most relevant items via iterative rounds of feedback from an expert panel. The first step was to identify and recruit a diverse group of international experts with relevant experience in LGBTQI+ health care, psychometric scale development, and clinical competence. These experts included health professionals (mostly physicians and psychologists), LGBTQI+ health advocates, clinicians, researchers, public health professionals with expertise in biostatistics and epidemiology, and educators. In total, the panel was comprised of 12 members. There were 3 Delphi rounds. In the first round of item generation, experts on the study team developed a broad number of items (100) both from the existing literature and newly formed items, which were then randomly listed and presented to the expert panel. Experts were asked to evaluate each item based on its relevance, clarity, and its potential importance for measuring LGBTQI+ related competencies among health care professionals. Experts could also comment on each item and suggest new or adapted items. After the first round, responses were scored for relevance and ordered accordingly. Quantitative consensus was defined a priori as a threshold of ≥75% and a median rating of ≥4 on a 5-point scale indicating an item as important or very important. These thresholds align with accepted Delphi standards and ensure that retained items reflect high agreement and rating stability. Based on the scoring (1-100), 39 items that showed strong consensus for inclusion were retained. These items are measured on a 5-point Likert-style scale that ranges from 1 (strongly disagree) to 5 (strongly agree).

In designing the scale, the team made several decisions to ensure applicability and interpretability. We drew upon existing implicit bias scales. While the scale is aimed toward those who provide clinical care, we ensured that the questions had a broad reach by using “clients or patients” instead of “patients” alone. We also avoided questions that were overly technical or related to specific specializations. While we believe it is important to address the needs of specific sexual and gender minority groups, we restricted these to a few questions rather than repeating questions by group. We elected to phrase questions in a way that addresses perceptions of one’s specific institution rather than perception of self. Finally, we avoided some questions over concerns about long-term relevance (eg, specifying pronouns when working with colleagues or clients, as this may become more normalized over time).

In addition to the 100 test items, to enable convergent construct validity testing and criterion and concurrent validity testing of the PRIDE scale, 2 further scales were used forming part of the main survey; these included the LGBT-DOCSS [22]; and the LGBTCI [24,28]. The LGBT-DOCSS is an internationally established, interdisciplinary self-assessment for health and mental health professionals working with LGBT clients [21]. The scale is comprised of 18-items and 3 subscales (clinical preparedness, attitudes, and knowledge). Items are rated on a 7-point Likert scale (1 strongly disagree to 7 strongly agree), with 8 items reverse-scored. For our purposes, we adjusted this to a 5-point scale, as we felt it would reduce cognitive load and potentially improve response rates. The loss of granularity would have minimal to no loss of psychometric information. From these items, a total mean score and 3 subscale scores (clinical preparedness, attitudes, and knowledge) can be computed. Higher scores thus reflect greater levels of LGBT cultural competence. The LGBT-DOCSS has demonstrated strong reliability and construct validity [22]. The LGBTCI is a well-used and researched assessment of LGBT workplace support and overall climate [24,28]. For this survey, we used only the 12-item Workplace Support subscale, in which items are rated on a 4-point Likert scale from 1 (doesn’t describe at all) to 4 (describes extremely well).

Additionally to the above, the main survey also included screening items (namely, consent, previous participation check, and age) and demographics (current employment, clinical and practice specialization, health and social care facility type, gender identity, sexual orientation, network diversity, ethnicity, and political ideology). The exact items and their phrasing were adjusted between the United States and European versions of the surveys to ensure cultural relevance.

### Design

A cross-sectional multicenter, multicountry scale validation study. Participants were recruited from October 2024 to December 2025. Study sites were located in Boston (United States), Washington, DC (United States), New York (United States), Brighton (United Kingdom), and Verona (Italy).

### Study Population and Sample

The study population consisted of health professionals, including students or health professionals-in-training, such as physicians, nurses, and other allied health professionals (eg, podiatrists, occupational therapists, physiotherapists, and mental health professionals) working in Italy, the United Kingdom, and the United States, with respondents able to complete the survey in English or Italian, depending on the country. The goal was to have a norming sample of international health care professionals including those in-training.

Sample size was calculated considering the 2 main statistical techniques to be used in the analysis: exploratory factor analysis (EFA) and confirmatory factor analysis (CFA). Based on the literature, as a rule of thumb (item-to-participant ratio), 5 to 10 participants per item are required [29-32]. For the 39-item PRIDE scale, this would translate to a minimum of 195 participants (5 participants per item) and a preferred sample size of 390 participants (10 participants per item). However, as we are planning an EFA and CFA, a total of approximately 700 participants is planned. For test-retest reliability, based on Walter et al [33], and assuming a null intraclass correlation coefficient of 0.40, an expected intraclass correlation coefficient of 0.70, 2 administrations of the scale, a significance level (α) of 0.05, and a power (1–β) of 0.80, a sample size of 62 is required.

### Inclusion and Exclusion Criteria

Eligible participants needed to be aged 18 years or older and be a health care professional or health care educator in a health and/or social care setting in Italy, the United Kingdom, or the United States (eg, physician, psychiatrist, psychologist, counselor, social worker, occupational therapist, university lecturer, paramedic, nongovernmental organization practitioner, nurse, etc) or currently training for a profession involving health and/or social care (eg, student nurse, medical student, mental health therapist-in-training); and be able to provide informed consent (online) to participate in the survey. Exclusion criteria were those aged under 18 years, and not a health care professional (or educator) or health care professional (or educator) in-training.

### The PRIDE Survey

**Figure 1.**
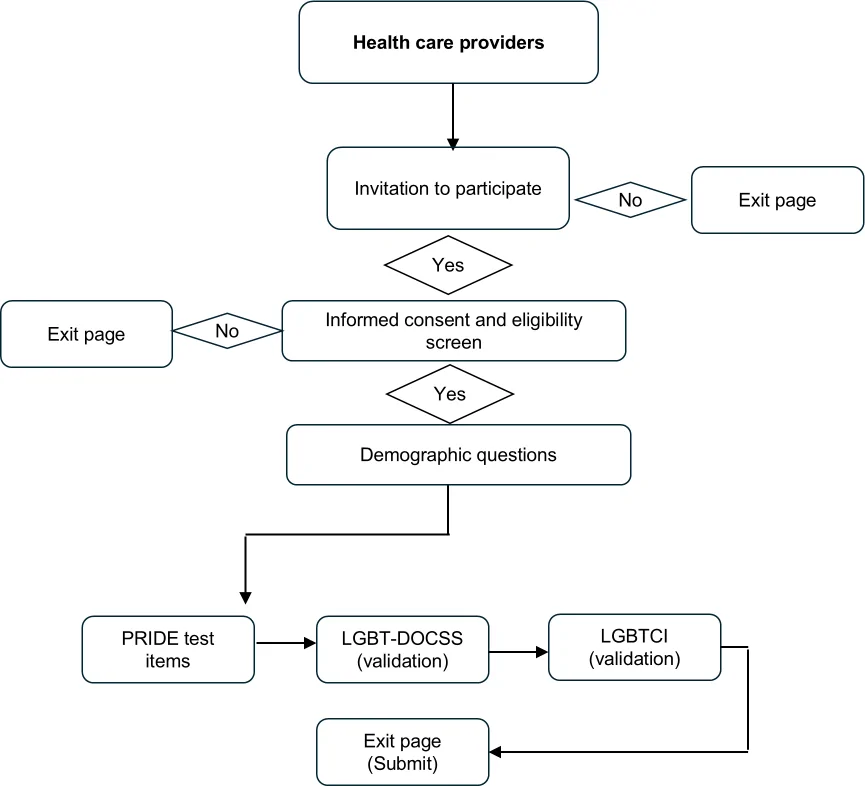
Flow diagram of the Professional Readiness in Diversity and Equity (PRIDE) questionnaire structure. LGBT-DOCSS: Lesbian, Gay, Bisexual, and Transgender Development of Clinical Skills Scale; LGBTCI: LGBT Climate Inventory.

The final PRIDE survey comprised three main sections as follows (Figure 1): (1) participant screening; (2) demographics; and (3) 39 test items, together with the LGBT-DOCSS [22] and 12 items from the LGBTCI scale [24]. The questionnaire items were translated and back translated for the Italian version. A copy of the final English version of the questionnaire is available as Multimedia Appendix 1. The protocol was developed and completed using the SPIROS (Standardized Protocol Items Recommendations for Observational Studies) guidelines (Checklist 1) [34].

### Study Procedure and Recruitment

#### Recruitment, Enrollment, and Consent

A convenience sampling approach was adopted and complemented by snowball recruitment. The survey link(s) and/or QR code was distributed via professional mailing lists, LGBTQI+ associations, and international conferences with recipients encouraged to forward it to colleagues to maximize dissemination for recruitment purposes. Additional routes included undergraduate and postgraduate teaching sessions providing recruitment opportunities for both the United States and European sites. Boston University (United States) used individualized links for medical school faculty, with other centers using an anonymous link (a common link for all participants from the same institution was created, and this link was different for each institution participating in the study).

The introductory page of the survey included detailed participation information and details of relevant support organizations for issues covered by the survey. Information was provided about the project, including details of what participants were required to do and how long it would take to complete the questions. A statement was provided regarding data protection, including confidentiality and anonymity. Potential participants were asked to click on a box to confirm that they had met the following criteria: (1) read and understood the participant information before proceeding, (2) understood that the survey was voluntary and that they agreed to participate, and (3) they were 18 years of age or older. There were minor variations in how this information was presented and/or its level of detail between European and US sites to allow for cultural variations and accepted practices within the guidance of the relevant institutional review boards (IRBs).

#### Data Management

The data collection was carried out using a General Data Protection Regulation (GDPR) [35] compliant platform. The REDCap (Vanderbilt University) platform [36,37] was hosted on secure institutional servers, ensuring compliance with the EU GDPR 2016‐679, United Kingdom Data Protection Act 2018 [38], and the US Common Rule Department of Health and Human Services 45 CFR 46 [39]. For the United Kingdom, REDCap v.16.0.15 was used; for the United States and Italy, REDCap v.16.1.6 was used.

Data were entered directly by participants through REDCap, thereby excluding data entry errors and transcription risks. All fields that formed part of the PRIDE scale (39 items) were set as required (to minimize incomplete data). Each record was automatically time-stamped with the date and time of completion. No personally identifiable information was stored alongside survey responses. Access to identifying information (eg, participant contact list) is restricted to authorized personnel and stored in a separate, secure REDCap instrument, linked only through coded identifiers.

Data quality checks were performed using REDCap’s Data Quality module and ad hoc statistical syntax scripts. Data will be exported in deidentified format to statistical software (Stata v.17) for psychometric and statistical analyses. Data export rights are restricted to authorized personnel. Variable names, labels, and coding schemes will follow the project’s codebook, ensuring reproducibility and interoperability across analysis phases.

### Statistical Analysis

#### Analytical Methods

**Figure 2.**
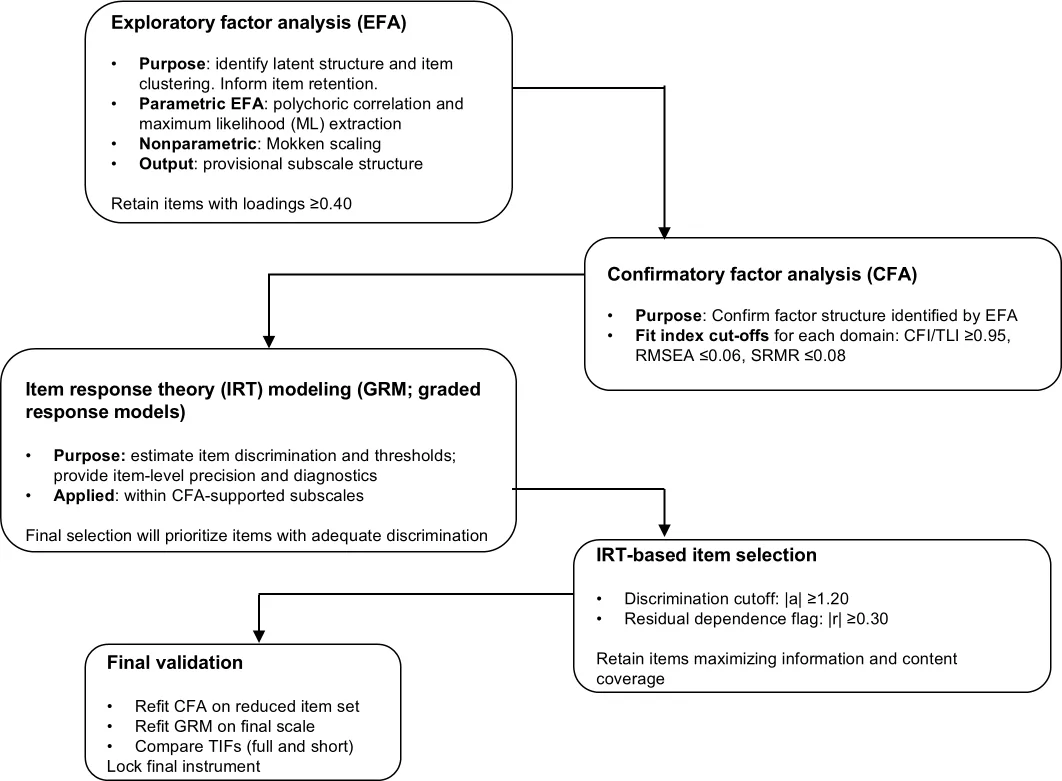
Professional Readiness in Diversity and Equity (PRIDE) scale statistical analysis plan for scale evaluation. CFI: comparative fit index; GRM: graded response model; ML: maximum likelihood; RMSEA: root mean square error of approximation; SRMR: standardized root mean square residual; TIFs: test information functions; TLI: Tucker-Lewis index.

All analyses will be performed using Stata (v.17) and R (v.4.5.1; R Foundation for Statistical Computing); Figure 2 shows the planned statistical analysis flowchart. We begin by analyzing the distributions of scale items. Continuous variables will be analyzed using descriptive statistics such as mean, median, standard deviation, quartiles, and IQRs, while for categorical variables, frequencies, proportions, or percentages will be used. Given the probable nonnormal distribution of items and scale scores, numerical comparisons will be performed using Dunn [40] test for stochastic dominance among multiple pairwise comparisons, following a Kruskal-Wallis test of stochastic dominance among k groups [41]. We will use EFA and CFA to identify dependence structures among measures, and item response theory (IRT) to explore factor diagnostics and retain items that carry the greatest exploratory power. Validation analyses will be conducted using the Kruskal-Wallis test with Bonferroni correction for multiple comparisons.

Missing data will be assessed prior to all analyses. For the United States sample, participants were required to fill all survey items, with branching (skip) logic implemented for respondents who did not currently engage in clinical practice. For the European sample, participants were permitted to skip responses for demographics only. We concatenated all complete and partial responses and participants with more than 10% of items missing will be excluded. For the remaining participants, missing values in the CFA framework will be handled using full information maximum likelihood estimation [42,43]. Descriptive and known-groups validity analyses will use pairwise deletion. The proportion and pattern of missing data will be reported, and missing completely at random test by Little will be used to evaluate the missing data mechanism.

As the use of convenience sampling (eg, through professional networks, institutional contacts, and online platforms) can introduce selection bias, to evaluate sample representativeness, the demographic profile of respondents in each country will be compared against available national health care workforce statistics. To ensure reproducibility of all data management and analytic procedures, a fixed random seed will be set prior to all data manipulation steps (seed=123,456). Prior to splitting, early respondents will be identified within each country as the first 25% of participants by enrollment order, to allow subsequent assessment of early responder bias. The sample will then be randomly split 50/50 within each country, with participant order randomized using a uniform random variable to remove any time- or motivation-related ordering effects, yielding Split A as the estimation subsample for EFA and Split B as the holdout subsample for CFA. Balance between the 2 subsamples will be verified for country distribution, LGBTQI+ identity status, and the proportion of early respondents, both overall and within each country, using cross-tabulations and Fisher exact tests. Limitations associated with convenience sampling, including potential volunteer bias and overrepresentation of professionals with prior interest in LGBTQI+ inclusive practice, will be explicitly acknowledged and considered in interpreting the generalizability of the validation findings.

#### EFA and CFA

EFA will be conducted to examine the underlying dimensional structure of the PRIDE scale and to inform item retention. As an initial parametric approach, EFA will be performed using principal component analysis (PCA) with varimax (orthogonal) rotation to obtain a simple and interpretable factor solution. Prior to extraction, the suitability of the data for factor analysis will be assessed (eg, inspection of the correlation matrix and standard measures of sampling adequacy). The number of factors to retain will be determined using the Kaiser criterion (eigenvalues ≥1) in conjunction with visual inspection of the scree plot to identify the point of inflection. Items will be evaluated based on their pattern of loadings, and only items with primary factor loadings ≥0.40 will be considered for inclusion. Items showing substantial cross-loadings (eg, ≥0.30 on more than 1 factor) or weak or unstable loadings will be reviewed for removal or revision, with decisions guided by both statistical evidence and conceptual coherence with the intended constructs. Given the ordinal nature of Likert-type responses, a complementary nonparametric ordinal approach will also be implemented. Specifically, a polychoric correlation matrix will be estimated and subjected to factor analysis with varimax rotation, applying the same factor-retention rules (eigenvalues ≥1 and scree plot) and item-selection criteria (primary loading ≥0.40, limited cross-loading). Consistency of the factor structure across the PCA-based and polychoric-based solutions will be examined to strengthen inferences regarding dimensionality and to ensure that the final factor model is robust to distributional assumptions. We acknowledge that PCA is technically a data-reduction technique that accounts for total item variance rather than modeling shared latent variance exclusively, and therefore does not constitute a latent variable model in the strict sense [30,44]. Its use here is pragmatic, consistent with its established role as an initial item-selection tool at the exploratory stage. The robustness of the dimensional solution will be evaluated formally through the planned cross-validation against the polychoric-based factor solution and, most importantly, through the subsequent CFA with maximum likelihood estimation, which will constitute the primary evidence for the latent structure of the PRIDE Scale.

CFA will be used to confirm the factor structure identified in the EFA. Model fit will be evaluated using the following indices: chi-square statistic, comparative fit index (CFI), root mean square error of approximation (RMSEA), standardized root mean square residual (SRMR), Tucker-Lewis index, Akaike information criterion, and Bayesian information criterion will be used following cutoff recommendations by Hu and Bentler [45,46].

To establish the cross-cultural and cross-demographic equivalence of the PRIDE Scale, formal measurement invariance testing will be conducted using multigroup confirmatory factor analysis. Following the sequential approach recommended by Vandenberg and Lance [47] and Putnick and Bornstein [48], configural invariance (same factor structure across groups), metric invariance (equal factor loadings), and scalar invariance (equal item intercepts) will be tested sequentially across two sets of planned comparisons: (1) Italian-speaking and English-speaking (United Kingdom and United States combined) subsamples, to establish cross-linguistic and cross-national measurement equivalence; and (2) LGBTQI+ and cisgender heterosexual participants, to evaluate whether the scale functions equivalently across groups that differ in sexual orientation and gender identity; a particularly important consideration given the nature of the construct being measured. Model comparisons will be evaluated using the chi-square difference test and changes in CFI (ΔCFI ≤−0.010) and RMSEA (ΔRMSEA ≤0.015) as criteria for invariance [49]. Additional subgroup analyses will be conducted to assess the potential influence of professional background (student vs trained practitioner) and gender on the validity of the CFA solution [50]. Where measurement invariance testing reveals a failure of scalar invariance for specific items, differential item functioning (DIF) analyses will be conducted as a planned follow-up to identify items performing differently across subgroups. DIF will be evaluated using ordinal logistic regression, which enables the detection of both uniform and nonuniform DIF for polytomous Likert-type items. Items exhibiting significant and practically meaningful DIF will be flagged for review during subsequent scale refinement.

#### IRT Modeling

Although CFA together with internal reliability tests can be more than sufficient to validate scales for use in research and evidence-based practice, IRT modeling can be a considerable complement when used with CFA [51]. In our planned analysis, an IRT approach to item reduction and final item selection will follow a staged workflow integrating Mokken scale analysis (MSA) as a nonparametric technique and parametric IRT graded response models (GRMs), supported by dimensionality assessment and local-dependence diagnostics [52,53]. MSA, together with the EFA results, will be used as the initial screening step to identify candidate item sets that demonstrate adequate scalability along a common latent trait and support the development of short forms with robust ordering properties. Specifically, scalability will be evaluated using Loevinger’s coefficients at the item level (Hi) and scale level (H), and reliability will be summarized [54]. Monotonicity and (where targeted) invariant item ordering will be examined using standard Mokken diagnostics to detect items with substantive violations (eg, nonmonotone response functions or marked ordering inconsistencies). Items showing weak scalability, notable monotonicity violations, or poor ordering behavior will be deprioritized, particularly when they provide redundant content relative to stronger items, while maintaining conceptual coverage across the domain.

GRM-based IRT will then be applied as the confirmatory and refinement step for the candidate item set(s) retained from MSA. Dimensionality will be evaluated to ensure each subscale is sufficiently unidimensional to support GRM estimation. Local item independence will be assessed via residual correlations (correlations among item residuals after accounting for the latent factor); item pairs showing elevated residual correlations will be interpreted as potential local dependence (eg, redundancy or shared wording) and will trigger content review, with prioritization of removing the weaker item. For each subscale, GRMs will be estimated to quantify item discrimination and category threshold functioning; item characteristic curves and test information functions will be used to evaluate category performance and the contribution of each item to measurement precision across the targeted trait range. Final selection will prioritize items with adequate discrimination, ordered and well-spaced thresholds, complementary information profiles, minimal local dependence, and preserved content balance.

#### Reliability Estimates and Test-Retest Reliability

Internal consistency reliability for the PRIDE scale will be evaluated within each sample (United States and Europe) and reported for each subscale, and for the total scale only if a defensible higher order or general-factor model supports total scoring. Reliability will be quantified using Cronbach α and, for ordinal Likert-type items, ordinal alpha (α_ord) computed from a polychoric correlation matrix. In addition, McDonald omega (ω) will be estimated for each monotone or unidimensional subscale using a congeneric factor model (allowing heterogeneous item loadings) and will be treated as the primary model-based estimate; where relevant, omega estimates derived under an ordinal framework (polychoric correlations or appropriate ordinal estimators) will be reported. Item-total correlations will be examined to assess the contribution of individual items to subscale reliability, and items with low item-total correlation (eg, <0.30) will be flagged for potential removal in conjunction with evidence from MSA, CFA (factor loadings or model fit), and IRT GRM outputs (discrimination, thresholds, and information), in accordance with the prespecified item selection rules. Test-retest reliability correlations will establish temporary (2-wk) stability of the PRIDE Scale using the blinded combined United States and European samples pooled. The test-retest sample size power calculation suggested an ideal sample size of 62 for this reliability test.

### Ethical Considerations

The following institutional review boards approved this study: University of Brighton Cross School Research Ethics Committee B (2025‐15162-Sherriff International LGBTQI+ competency scale); Verona University Hospital (PRIDE - Prog. 491CET); George Washington University (NCR246211, Pratt-Chapman Updating measurement for LGBTQI-Affirming Care); Boston University Medical Center (H-43531, Zumwalt Assessment of educator knowledge and attitudes about sexual and gender minority topics); City University of New York (2024‐0850-Hunter, Bidell Developing a New LGBTQI+ Clinical Competency Assessment Scale for Health and Mental Health Providers). Participants provided informed consent to participate, including an understanding involvement was voluntary, could be withdrawn at any time without justification, and that all information would be handled confidentially.

## Results

Data collection funded in May 2023 and was conducted from October 2024 to December 2025, with a total of 1339 eligible participants who completed the survey. Data cleaning and preparation are underway, and data analysis will take place in Summer 2026, with the first results paper expected to be submitted in Autumn 2026.

## Discussion

### Principal Findings

The development and validation of the new PRIDE scale will represent a significant advancement toward improved cultural competence among health care professionals in Europe and the United States. Validating the scale in English- and Italian-speaking populations will support the development of international interventions and, ultimately, treatment outcomes for LGBTQI+ people globally.

The PRIDE Scale is anticipated to make several distinct contributions to the field of LGBTQI+ health and to the broader science of clinical competency assessment. First, it will provide a contemporary, psychometrically robust instrument that reflects current language, conceptual nuance, and lived realities of LGBTQI+ communities, addressing well-documented limitations of existing tools. Currently, the most frequently used measure of health care professionals’ knowledge and attitudes toward LGBTQI+ individuals is the LGBT-DOCSS, developed nearly a decade ago [21,22], which no longer fully reflects contemporary language, concepts, or understandings of LGBTQI+ identities. Due to evolving cultural norms, some of the LGBT-DOCSS terminology is now perceived as outdated and, in some cases, offensive by survey-takers. The PRIDE Scale will enable researchers to build on the contributions of the LGBT-DOCSS while modernizing the language and explicitly incorporating perceptions of workplace climate, which has been shown to shape patient outcomes [55]. Second, by being developed and validated simultaneously across 3 countries and 2 languages, the PRIDE Scale is positioned as one of the few internationally validated LGBTQI+ competency measures, with formal measurement invariance testing supporting cross-cultural comparability. Third, by integrating individual-level competence (knowledge, attitudes, awareness, and skills) with organizational climate, the scale operationalizes a more ecological understanding of clinical care, consistent with growing evidence that workplace context shapes health care professional behavior and patient experience [25-27]. Fourth, the instrument is expected to function both as a research outcome measure (eg, for educational and organizational interventions) and as a practical self-assessment and quality-improvement tool in clinical and training settings.

Despite challenging political climates, health care professionals remain ethically bound to provide competent and compassionate care to all patients, including LGBTQI+ people. Yet, as social and cultural norms continue to shift, health care professionals are exposed to a wide range of messages about LGBTQI+ communities, starting in their youth, through their specific training experiences, and beyond during their practice as clinicians. These influences create and shape explicit and implicit biases among health care professionals which can negatively impact clinical competence and services for LGBTQI+ people [22]. Although efforts have been made to address such biases, including increasing the number of training hours devoted to LGBTQI+ health, accurately assessing health care professionals’ competence requires reliable and valid self-assessment tools [12,56].

### Limitations

Several limitations should be acknowledged. First, the convenience sampling approach, supplemented by snowball recruitment through professional mailing lists, LGBTQI+ associations, conferences, and institutional teaching sessions, limits the generalizability of the findings to the broader health care workforce in Italy, the United Kingdom, and the United States. To partially mitigate this, the sample demographics will be benchmarked against national statistics. Relatedly, the recruitment strategy is susceptible to network bias: health care professionals with preexisting interest in LGBTQI+ health may be overrepresented, while less engaged or less supportive professionals may be underrepresented, potentially attenuating variability in key constructs and inflating estimates of health care professional readiness and of an inclusive climate. The reliance on self-report measures further introduces social desirability bias, as participants may respond in ways that align with professional norms rather than their actual beliefs, knowledge, or behaviors; anonymous administration reduces but does not eliminate this concern.

Second, although the instrument was developed and administered in multiple languages across 3 national contexts, translation and cross-cultural adaptation processes inevitably introduce subtle semantic and conceptual differences across versions. While measurement invariance testing and Differential Item Functioning analyses will identify items that function differently across linguistic, cultural, or demographic groups, some degree of construct nonequivalence may persist, particularly given the heterogeneous legal, cultural, and clinical frameworks governing LGBTQI+ health across the participating countries.

Third, the Delphi expert panel, while interdisciplinary and international, comprised 12 members, and consensus-based item generation is inherently shaped by the perspectives, disciplinary backgrounds, and sociocultural positioning of the panelists. Although the 75 percent consensus threshold and 3-round structure were designed to enhance rigor, alternative panel compositions might have yielded different item pools, particularly regarding emerging or contested constructs within LGBTQI+ health.

Finally, the PRIDE Scale captures health care professional–reported competence and perceived workplace climate but does not incorporate patient-reported experiences or objective behavioral indicators of inclusive care. Triangulation with patient outcomes, simulated clinical encounters, or organizational audit data will be necessary in future work to fully establish the criterion validity and real-world utility of the instrument. Future longitudinal work will be required to establish these properties and to assess the scale’s responsiveness to educational or organizational interventions.

### Conclusions

The development and validation of the new PRIDE scale will represent a significant advancement toward improved cultural competence among health care professionals. Validating the scale in English and Italian-speaking populations will support the development of international interventions and ultimately improve treatment outcomes for LGBTQI+ individuals. Just as the 2017 LGBT-DOCSS has been used to assess medical students [57], residents [58], social workers [59], and interprofessional teams in oncology centers [60], the PRIDE Scale is expected to be used in a myriad of clinical and public health settings. Consequently, the PRIDE Scale has the potential to have a far-reaching impact on the provision of clinical care and public health services across the globe.

## Supplementary material

10.2196/95456 Multimedia Appendix 1 Final version of questionnaire (English).

10.2196/95456 Checklist 1 SPIROS checklist.

## Abbreviations

CFA: confirmatory factor analysis
CFI: comparative fit index
DIF: differential item functioning
EFA: exploratory factor analysis
GDPR: General Data Protection Regulation
GRM: graded response model
IRB: institutional review board
IRT: item response theory
LGBT-DOCSS: Lesbian, Gay, Bisexual, and Transgender Development of Clinical Skills Scale
LGBTCI: LGBT Climate Inventory
LGBTQI+: lesbian, gay, bisexual, transgender, queer, and intersex
MSA: Mokken scale analysis
PCA: principal component analysis
QUIRKS: Queering Individual Relational and Knowledge Scales
RMSEA: root mean square error of approximation
SPIROS: Standardized Protocol Items Recommendations for Observational Studies
SRMR: standardized root mean square residual
TIF: test information functions
