# Supplementary material for: Development and Validation of the Professional Readiness in Diversity and Equity Scale: Protocol for a Scale Validation Study
Source: JMIR Res Protoc. 2026 Sep 21;15:e95456. doi: 10.2196/95456 (PMC13592149; doi:10.2196/95456)
Supplement: Supplementary file 1 [file resprot-v15-e95456-s001.pdf]

Data Dictionary Codebook

	#	Variable / Field Name	Field Label<br>*Field Note*	Field Attributes (Field Type, Validation, Choices, Calculations, etc.)
**Instrument: PRIDE Scale (UK version) (pride_scale_uk_version)** Enabled as survey				
	1	[record_id]	Record ID	text
	2	[consent]	Do you consent to participate in this survey?	yesno, Required<br>1 Yes<br>0 No<br><br>Stop actions on 0
	3	[duplicate]	Have you already participated in this survey?	yesno, Required<br>1 Yes<br>0 No<br><br>Stop actions on 1
	4	[age]	How old are you?	text (number, Min: 0, Max: 99), Required<br>Custom alignment: LV
	5	[student_status]<br><br>Show the field ONLY if:<br>[age] >= 18	Section Header: *Let's start with a few simple questions about you... As this is a pilot survey, we are looking to test and improve some of these questions which are drawn from existing validated questionnaires. Some questions may therefore feel repetitive or use out-dated language. Please be assured they are part of our research design so do answer them if you feel able.*<br><br>Are you a student or professional in-training?	radio<br>1 Yes<br>2 No
	6	[student_role]<br><br>Show the field ONLY if:<br>[student_status] = '1'	How would you best describe your role?	radio<br>1 Medical Student<br>2 Doctor in Training (e.g., Foundation Year, Residency)<br>3 Nurse student<br>4 Social Worker student<br>11 Psychology student<br>5 Occupational Therapist student<br>6 Paramedic student<br>7 Physiotherapist student<br>8 Radiographer student<br>9 Podiatrist student

				10 Other (Allied) Healthcare Professional {prof_role_plse_specify}<br><br>Custom alignment: LV
	7	[prof_role_plse_specify]<br><br>Show the field ONLY if:<br>[student_role] = '10'	Please specify	text<br>Field Annotation: @PLACEHOLDER='Please specify:'
	8	[professional_role]<br><br>Show the field ONLY if:<br>[student_status] = '2'	How would you describe your current profession or position?	radio<br>1 Doctor (e.g. General Practitioner, family doctor)<br>2 Consultant/Specialist<br>3 Nurse<br>4 Social Worker<br>11 Psychologist<br>5 Occupational Therapist<br>6 Paramedic<br>7 Physiotherapist<br>8 Radiographer<br>9 Podiatrist<br>10 Other (Allied) Healthcare Professional {prof_role_plse_specify_2}<br><br>Custom alignment: LV
	9	[prof_role_plse_specify_2]<br><br>Show the field ONLY if:<br>[professional_role] = '10'	Please specify	text<br>Field Annotation: @PLACEHOLDER='Please specify:'
	10	[clinical_experience]<br><br>Show the field ONLY if:<br>[age] >= 18	How many years of clinical and/or professional experience do you have? (please include any student placement or practice experience)	radio<br>1 None<br>2 Less than 2 years<br>3 2-5 years<br>4 6-10 years<br>5 More than 10 years
	11	[speciality]<br><br>Show the field ONLY if:<br>[age] >= 18	What is your area of specialty or clinical practice (if applicable)	radio<br>0 None at the moment<br>1 General Practice/Primary Care<br>2 Internal Medicine

				<table><tr><td>3</td><td>Surgery</td></tr><tr><td>4</td><td>Paediatrics</td></tr><tr><td>5</td><td>Psychiatry/Mental Health</td></tr><tr><td>6</td><td>Obstetrics & Gynaecology</td></tr><tr><td>7</td><td>Emergency Medicine</td></tr><tr><td>8</td><td>Community Health</td></tr><tr><td>9</td><td>Public Health</td></tr><tr><td>10</td><td>Social Work</td></tr><tr><td>11</td><td>Other {speciality_plse_specify}</td></tr></table><br>Custom alignment: LV<br>Field Annotation:<br>@PLACEHOLDER='Please specify:'
	12	[speciality_plse_specify]<br>Show the field ONLY if:<br>[speciality] = '11'	Please specify	text<br>Field Annotation:<br>@PLACEHOLDER='Please specify:'
	13	[demo_race]<br>Show the field ONLY if:<br>[age] >= 18	What is your ethnic group?	radio<br><table><tr><td>11</td><td>Asian or Asian British</td></tr><tr><td>12</td><td>Black, Black British, Caribbean or African</td></tr><tr><td>13</td><td>Mixed / Multiple ethnic groups</td></tr><tr><td>14</td><td>Other ethnic group</td></tr><tr><td>15</td><td>White</td></tr><tr><td>16</td><td>Self-identify {demo_race_spc}</td></tr><tr><td>-9</td><td>Prefer not to answer</td></tr></table><br>Custom alignment: LV<br>Field Annotation:<br>@NONEOFTHEABOVE='-9'
	14	[demo_race_spc]<br>Show the field ONLY if:<br>[demo_race]=16	Specify ethnicity	text<br>Custom alignment: LV<br>Field Annotation:<br>@PLACEHOLDER='Please specify:'
	15	[demo_gender]<br>Show the field ONLY if:<br>[age] >= 18	Which best describes your sex at birth?	radio, Required<br><table><tr><td>1</td><td>Female</td></tr><tr><td>2</td><td>Intersex</td></tr><tr><td>3</td><td>Male</td></tr><tr><td>-9</td><td>Prefer not to answer</td></tr></table><br>Custom alignment: LV<br>Field Annotation:<br>@NONEOFTHEABOVE='-8, -9'

<table>
<tr><td></td><td>16</td><td>[demo_sex]

Show the field ONLY if:
[age] >= 18</td><td>Is the gender you identify with the same as your sex registered at birth?</td><td>radio, Required
1 Yes
2 No (please specify): {gender_id_spc}
3 Prefer not to say

Custom alignment: LV
Field Annotation: @NONEOFTHEABOVE='3'</td></tr>
<tr><td></td><td>17</td><td>[gender_id_spc]

Show the field ONLY if:
[demo_sex] = '2'</td><td>Please specify (e.g. non-binary, transmasculine etc)</td><td>text
Custom alignment: LV</td></tr>
<tr><td></td><td>18</td><td>[demo_orient]

Show the field ONLY if:
[age] >= 18</td><td>Which of the following best describes your sexual orientation?</td><td>radio, Required
1 Heterosexual or 'straight'
2 Lesbian or gay
3 Bisexual
4 Asexual
9 Use a different term: {demo_orient_spc}
-8 Don't know
-9 Prefer not to answer

Custom alignment: LV
Field Annotation: @NONEOFTHEABOVE='-9'</td></tr>
<tr><td></td><td>19</td><td>[demo_orient_spc]

Show the field ONLY if:
[demo_orient]=9</td><td>Specify sexual orientation</td><td>text
Custom alignment: LV</td></tr>
<tr><td></td><td>20</td><td>[demo_politics]

Show the field ONLY if:
[age] >= 18</td><td>Using the following scale, what best describes your political (or social) ideology?</td><td>radio
2 Liberal
3 Moderate
4 Conservative
-8 Unsure/do not know
-9 Prefer not to answer

Custom alignment: LV
Field Annotation: @NONEOFTHEABOVE='-9'</td></tr>
<tr><td></td><td>21</td><td>[progress_1]</td><td>Survey 33% Complete</td><td>descriptive</td></tr>
<tr><td></td><td>22</td><td>[net_work]

Show the field ONLY if:
[age] >= 18</td><td>Section Header: LGBTQI+ populations are defined as including, but not limited to, individuals who identify as lesbian, gay, bisexual, asexual, transgender, Two-Spirit, queer, and/or intersex. Individuals with same-sex or -gender attractions</td><td>radio
0 None
1 1-5</td></tr>
</table>

			or behaviours and those with a difference in sex development are also included.<br>Think about 20 individuals within your workplace (practice or placement) with whom you've interacted most frequently in the past three months. To the best of your knowledge, how many of these individuals identify as LGBTQI+ ?	2 6-10<br>3 11-15<br>4 16-20<br>-8 Unsure/Do not know<br>-9 Prefer not to answer<br>Custom alignment: LV<br>Field Annotation: @NONEOFTHEABOVE='-9'
	23	[net_friendfam]<br>Show the field ONLY if:<br>[age] >= 18	Think about the 20 friends and family members with whom you've interacted most frequently in the past three months. To the best of your knowledge, how many of these individuals identify as LGBTQI+ ?	radio<br>0 None<br>1 1-5<br>2 6-10<br>3 11-15<br>4 16-20<br>-8 Unsure/Do not know<br>-9 Prefer not to answer<br>Custom alignment: LV<br>Field Annotation: @NONEOFTHEABOVE='-9'
	24	[clin_head]<br>Show the field ONLY if:<br>[age] >= 18	The following questions will address the characteristics of the clinical care you provide.	descriptive
	25	[clin_loc]<br>Show the field ONLY if:<br>[age] >= 18	What is the location of your clinical practice or placement? Choose all that apply.	checkbox<br>1 clin_loc___1 Hospital<br>2 clin_loc___2 Community<br>3 clin_loc___3 Private practice<br>4 clin_loc___4 Other: {clin_loc_spc}<br>5 clin_loc___5 Do not practice/Do not currently practice.<br>Custom alignment: LV<br>Field Annotation: @NONEOFTHEABOVE='5'
	26	[clin_loc_spc]<br>Show the field ONLY if:<br>[clin_loc(4)] = '1'	Specify location of clinical practice or placement	text<br>Custom alignment: LV<br>Field Annotation: @PLACEHOLDER='Please specify:'
	27	[clin_patients]<br>Show the field ONLY if:	In an average week of clinical practice or placement in the last three months, how many patients do you see?	text<br>Custom alignment: LV

		[clin_loc(1)] = '1' or [clin_loc(2)] = '1' or [clin_loc(4)] = '1' or [clin_loc(3)] = '1'		
	28	[urban_rural]<br><br>Show the field ONLY if:<br>[clin_loc(1)] = '1' and [clin_loc(2)] = '1' and [clin_loc(3)] = '1' and [clin_loc(4)] = '1'	Are these locations mostly?	radio<br>1 Urban<br>2 Rural<br>3 Mixed<br><br>Custom alignment: LV
	29	[clin_diverse]<br><br>Show the field ONLY if:<br>[clin_loc(1)] = '1' or [clin_loc(2)] = '1' or [clin_loc(4)] = '1' or [clin_loc(3)] = '1'	Please rate your agreement with the following statement: The patients I care for are diverse with respect to individuals' sexual orientations and/or gender identities.	radio<br>1 Strongly agree<br>2 Agree<br>3 Neither agree nor disagree<br>4 Disagree<br>5 Strongly disagree<br>-8 Unsure/Do not know<br>-9 Prefer not to answer<br><br>Custom alignment: LV<br>Field Annotation: @NONEOFTHEABOVE='-9'
	30	[formal_training]<br><br>Show the field ONLY if:<br>[age] >= 18	Have you received formal training on LGBTQI+ healthcare issues?	radio<br>1 Yes<br>2 No<br>3 Unsure<br><br>Custom alignment: LV
	31	[hours_training]<br><br>Show the field ONLY if:<br>[formal_training] = '1'	Approximately, how many hours did this training involve?	radio<br>0 None<br>1 1-2 hours<br>2 3-5 hours<br>3 6-8 hours<br>4 9 hours or more<br><br>Custom alignment: LV
	32	[perc_noneedtoknow]<br><br>Show the field ONLY if:<br>[age] >= 18	Section Header: *Instructions: Items on this scale are intended to examine preparedness, attitudes, and knowledge regarding lesbian, gay, bisexual, transgender, queer, intersex, and other sexual minority (LGBTQI+) people. Please use the provided scale to rate your level of agreement or disagreement for each item where 1=strongly disagree, 2=disagree, 3= Neither agree nor disagree, 4 = agree, 5=strongly agree. Note that you will complete two validated survey instruments. Some items may feel repetitive due to the nature of the validation process. Some*	radio (Matrix), Required<br>1 Strongly disagree1<br>2 2<br>3 Neither agree nor disagree3<br>4 4<br>5 Strongly agree5

			questions may use strong language that some may find outdated or upsetting. Please answer if you feel able as we will use the findings to improve question wording & inform future research & training of health & social care professionals.<br>I treat all my clients/patients the same, so I do not need to know their sexual orientation or gender identity.	
	33	[perc_duty]<br>Show the field ONLY if:<br>[age] >= 18	Healthcare providers have a duty to care for LGBTQI+ clients/ patients.	radio (Matrix), Required<br>1 Strongly disagree1<br>2 2<br>3 Neither agree nor disagree3<br>4 4<br>5 Strongly agree5
	34	[perc_disclose]<br>Show the field ONLY if:<br>[age] >= 18	Client/ patient forms should provide ways to disclose sexual orientation and gender identity data.	radio (Matrix), Required<br>1 Strongly disagree1<br>2 2<br>3 Neither agree nor disagree3<br>4 4<br>5 Strongly agree5
	35	[perc_marriage]<br>Show the field ONLY if:<br>[age] >= 18	Same-sex marriage is important recognition of a committed relationship.	radio (Matrix), Required<br>1 Strongly disagree1<br>2 2<br>3 Neither agree nor disagree3<br>4 4<br>5 Strongly agree5
	36	[perc_notknownlgbt]<br>Show the field ONLY if:<br>[age] >= 18	I would not want to be recognised professionally as a provider that knowingly cares for LGBTQI+ clients/patients.	radio (Matrix), Required<br>1 Strongly disagree1<br>2 2<br>3 Neither agree nor disagree3<br>4 4<br>5 Strongly agree5
	37	[perc_committed]<br>Show the field ONLY if:<br>[age] >= 18	Same-sex relationships are as committed as cisgender, heterosexual relationships.	radio (Matrix), Required<br>1 Strongly disagree1<br>2 2<br>3 Neither agree nor disagree3<br>4 4<br>5 Strongly agree5
	38	[perc_normal]<br>Show the field ONLY if:<br>[age] >= 18	Same-sex attraction is a normal part of the human experience.	radio (Matrix), Required<br>1 Strongly disagree1<br>2 2

				3 Neither agree nor disagree3<br>4 4<br>5 Strongly agree5
	39	[perc_trauma]<br>Show the field ONLY if:<br>[age] >= 18	Many LGBTQI+ people experience significant trauma over their lifespan.	radio (Matrix), Required<br>1 Strongly disagree1<br>2 2<br>3 Neither agree nor disagree3<br>4 4<br>5 Strongly agree5
	40	[perc_blocker]<br>Show the field ONLY if:<br>[age] >= 18	Healthcare providers should be able to provide young people under the age of 18 with hormone blockers.	radio (Matrix), Required<br>1 Strongly disagree1<br>2 2<br>3 Neither agree nor disagree3<br>4 4<br>5 Strongly agree5
	41	[perc_imptoknow]<br>Show the field ONLY if:<br>[age] >= 18	It's important to know the sexual orientation and gender identity of my clients/ patients when treating their physical and mental health concerns.	radio (Matrix), Required<br>1 Strongly disagree1<br>2 2<br>3 Neither agree nor disagree3<br>4 4<br>5 Strongly agree5
	42	[perc_competant]<br>Show the field ONLY if:<br>[age] >= 18	I am competent addressing the health needs of LGBTQI+ clients/ patients.	radio (Matrix), Required<br>1 Strongly disagree1<br>2 2<br>3 Neither agree nor disagree3<br>4 4<br>5 Strongly agree5
	43	[perc_comfortaround]<br>Show the field ONLY if:<br>[age] >= 18	I am comfortable around LGBTQI+ people that differ from conventional gender norms.	radio (Matrix), Required<br>1 Strongly disagree1<br>2 2<br>3 Neither agree nor disagree3<br>4 4<br>5 Strongly agree5
	44	[perc_uncomfortable]<br>Show the field ONLY if:<br>[age] >= 18	I am uncomfortable working with LGBTQI+ clients/ patients.	radio (Matrix), Required<br>1 Strongly disagree1<br>2 2<br>3 Neither agree nor disagree3<br>4 4

				5 Strongly agree5
	45	[perc_comforttreat]<br>Show the field ONLY if:<br>[age] >= 18	I am comfortable treating LGBTQI+ clients/ patients.	radio (Matrix), Required<br>1 Strongly disagree1<br>2 2<br>3 Neither agree nor disagree3<br>4 4<br>5 Strongly agree5
	46	[perc_accepting]<br>Show the field ONLY if:<br>[age] >= 18	Overall, my colleagues are accepting of LGBTQI+ employees.	radio (Matrix), Required<br>1 Strongly disagree1<br>2 2<br>3 Neither agree nor disagree3<br>4 4<br>5 Strongly agree5
	47	[perc_challengdisc]<br>Show the field ONLY if:<br>[age] >= 18	When I witness LGBTQI+ discrimination in my workplace, I actively challenge it.	radio (Matrix), Required<br>1 Strongly disagree1<br>2 2<br>3 Neither agree nor disagree3<br>4 4<br>5 Strongly agree5
	48	[perc_antidisc]<br>Show the field ONLY if:<br>[age] >= 18	There are clear LGBTQI+ anti-discrimination policies in my workplace.	radio (Matrix), Required<br>1 Strongly disagree1<br>2 2<br>3 Neither agree nor disagree3<br>4 4<br>5 Strongly agree5
	49	[perc_supportive]<br>Show the field ONLY if:<br>[age] >= 18	My work environment is a supportive place for LGBTQI+ people.	radio (Matrix), Required<br>1 Strongly disagree1<br>2 2<br>3 Neither agree nor disagree3<br>4 4<br>5 Strongly agree5
	50	[perc_negcomment]<br>Show the field ONLY if:<br>[age] >= 18	I have overheard negative LGBTQI+ comments from people I work with.	radio (Matrix), Required<br>1 Strongly disagree1<br>2 2<br>3 Neither agree nor disagree3<br>4 4<br>5 Strongly agree5

	51	[perc_worrylang]<br><br>Show the field ONLY if:<br>[age] >= 18	In my work environment, I am worried about using the wrong language regarding LGBTQI+ people.	radio (Matrix), Required<br>1 Strongly disagree1<br>2 2<br>3 Neither agree nor disagree3<br>4 4<br>5 Strongly agree5
	52	[perc_respect]<br><br>Show the field ONLY if:<br>[age] >= 18	My employer treats LGBTQI+ employees with respect.	radio (Matrix), Required<br>1 Strongly disagree1<br>2 2<br>3 Neither agree nor disagree3<br>4 4<br>5 Strongly agree5
	53	[perc_worryjob]<br><br>Show the field ONLY if:<br>[age] >= 18	I know colleagues that worry about job security because they identify as LGBTQI+.	radio (Matrix), Required<br>1 Strongly disagree1<br>2 2<br>3 Neither agree nor disagree3<br>4 4<br>5 Strongly agree5
	54	[perc_employeeout]<br><br>Show the field ONLY if:<br>[age] >= 18	It's easy for LGBTQI+ employees to be out where I work.	radio (Matrix), Required<br>1 Strongly disagree1<br>2 2<br>3 Neither agree nor disagree3<br>4 4<br>5 Strongly agree5
	55	[perc_negwork]<br><br>Show the field ONLY if:<br>[age] >= 18	The place where I work is negative for LGBTQI+ employees.	radio (Matrix), Required<br>1 Strongly disagree1<br>2 2<br>3 Neither agree nor disagree3<br>4 4<br>5 Strongly agree5
	56	[perc_referral]<br><br>Show the field ONLY if:<br>[age] >= 18	I am aware of referral pathways to meet the needs of my LGBTQI+ clients/ patients.	radio (Matrix), Required<br>1 Strongly disagree1<br>2 2<br>3 Neither agree nor disagree3<br>4 4<br>5 Strongly agree5
	57	[perc_specneed]<br><br>Show the field ONLY if:	LGBTQI+ people may have specific health (physical and/ or mental health)	radio (Matrix), Required<br>1 Strongly disagree1

		[age] >= 18	needs that differ from those of cisgender, heterosexual people.	2 2<br>3 Neither agree nor disagree3<br>4 4<br>5 Strongly agree5
	58	[perc_equalaccess]<br>Show the field ONLY if:<br>[age] >= 18	The LGBTQI+ and cisgender, heterosexual populations have equal access to healthcare.	radio (Matrix), Required<br>1 Strongly disagree1<br>2 2<br>3 Neither agree nor disagree3<br>4 4<br>5 Strongly agree5
	59	[perc_stress]<br>Show the field ONLY if:<br>[age] >= 18	As a group, LGBTQI+ people have higher levels of stress compared to cisgender, heterosexual people.	radio (Matrix), Required<br>1 Strongly disagree1<br>2 2<br>3 Neither agree nor disagree3<br>4 4<br>5 Strongly agree5
	60	[perc_intersect]<br>Show the field ONLY if:<br>[age] >= 18	When LGBTQI+ people are part of multiple marginalised groups, their experience of discrimination and stress may be significantly magnified.	radio (Matrix), Required<br>1 Strongly disagree1<br>2 2<br>3 Neither agree nor disagree3<br>4 4<br>5 Strongly agree5
	61	[perc_uniquerisk]<br>Show the field ONLY if:<br>[age] >= 18	LGBTQI+ people have unique healthcare risks and vulnerabilities.	radio (Matrix), Required<br>1 Strongly disagree1<br>2 2<br>3 Neither agree nor disagree3<br>4 4<br>5 Strongly agree5
	62	[perc_lgbttrain]<br>Show the field ONLY if:<br>[age] >= 18	I seek out LGBTQI+ specific training.	radio (Matrix), Required<br>1 Strongly disagree1<br>2 2<br>3 Neither agree nor disagree3<br>4 4<br>5 Strongly agree5
	63	[perc_rectrain]<br>Show the field ONLY if:<br>[age] >= 18	I have received adequate training to work effectively with LGBTQI+ clients/patients.	radio (Matrix), Required<br>1 Strongly disagree1<br>2 2<br>3 Neither agree nor disagree3

				4 4<br>5 Strongly agree5
	64	[perc_benefittrain]<br>Show the field ONLY if:<br>[age] >= 18	I would benefit from more LGBTQI+ training.	radio (Matrix), Required<br>1 Strongly disagree1<br>2 2<br>3 Neither agree nor disagree3<br>4 4<br>5 Strongly agree5
	65	[perc_nottrain]<br>Show the field ONLY if:<br>[age] >= 18	I don't feel adequately trained to work with LGBTQI+ clients/ patients.	radio (Matrix), Required<br>1 Strongly disagree1<br>2 2<br>3 Neither agree nor disagree3<br>4 4<br>5 Strongly agree5
	66	[perc_preptrans]<br>Show the field ONLY if:<br>[age] >= 18	I feel prepared to meet the health and/ or mental health needs of transgender and gender diverse people.	radio (Matrix), Required<br>1 Strongly disagree1<br>2 2<br>3 Neither agree nor disagree3<br>4 4<br>5 Strongly agree5
	67	[perc_prepintersex]<br>Show the field ONLY if:<br>[age] >= 18	I feel prepared to meet the health and/ or mental health needs of intersex people.	radio (Matrix), Required<br>1 Strongly disagree1<br>2 2<br>3 Neither agree nor disagree3<br>4 4<br>5 Strongly agree5
	68	[perc_prepgay]<br>Show the field ONLY if:<br>[age] >= 18	I feel prepared to meet the health and/ or mental health needs of gay, lesbian, and bisexual people.	radio (Matrix), Required<br>1 Strongly disagree1<br>2 2<br>3 Neither agree nor disagree3<br>4 4<br>5 Strongly agree5
	69	[perc_moral]<br>Show the field ONLY if:<br>[age] >= 18	I am morally uncomfortable with LGBTQI+ people.	radio (Matrix), Required<br>1 Strongly disagree1<br>2 2<br>3 Neither agree nor disagree3<br>4 4<br>5 Strongly agree5

	70	[perc_nounderstand]<br>Show the field ONLY if:<br>[age] >= 18	I don't understand transgender or gender diverse persons.	radio (Matrix), Required<br>1 Strongly disagree1<br>2 2<br>3 Neither agree nor disagree3<br>4 4<br>5 Strongly agree5
	71	[progress_2]	Survey 66% Complete	descriptive
	72	[docss_transbar]<br>Show the field ONLY if:<br>[age] >= 18	Section Header: *Knowledge, Attitudes and Behaviors (KAB) Instructions: Items on this scale are intended to examine preparedness, attitudes, and basic knowledge regarding lesbian, gay, bisexual, and transgender (LGBT) clients/patients. Please use the provided scale to rate your level of agreement or disagreement for each item where 1=strongly disagree to 5=strongly agree. Please note, items on this scale primarily enquire about either sexual orientation (LGB = lesbian, gay, and bisexual) or gender identity (transgender). Two questions are inclusive and refer collectively to lesbian, gay, bisexual, and transgender (LGBT) clients/patients.*<br>I am aware of institutional barriers that may inhibit transgender people from using healthcare services.	radio (Matrix), Required<br>1 Strongly disagree1<br>2 2<br>3 Neither agree nor disagree3<br>4 4<br>5 Strongly agree5
	73	[docss_lgbbar]<br>Show the field ONLY if:<br>[age] >= 18	I am aware of institutional barriers that may inhibit LGB people from using health services.	radio (Matrix), Required<br>1 Strongly disagree1<br>2 2<br>3 Neither agree nor disagree3<br>4 4<br>5 Strongly agree5
	74	[docss_transdisorder]<br>Show the field ONLY if:<br>[age] >= 18	I think being transgender is a mental disorder.	radio (Matrix), Required<br>1 Strongly disagree1<br>2 2<br>3 Neither agree nor disagree3<br>4 4<br>5 Strongly agree5
	75	[docss_unprep]<br>Show the field ONLY if:<br>[age] >= 18	I would feel unprepared talking with an LGBT client/patient about issues related to their sexual orientation or gender identity.	radio (Matrix), Required<br>1 Strongly disagree1<br>2 2<br>3 Neither agree nor disagree3<br>4 4<br>5 Strongly agree5
	76	[docss_ssnotcommit]<br>Show the field ONLY if:	A same sex relationship between two men or two women is not as strong and committed as one between a man and a woman.	radio (Matrix), Required<br>1 Strongly disagree1<br>2 2

		[age] >= 18		3 Neither agree nor disagree3<br>4 4<br>5 Strongly agree5
	77	[docss_lgbmoreprob]<br>Show the field ONLY if:<br>[age] >= 18	I am aware of research indicating that LGB individuals experience disproportionate levels of health and mental health problems compared to heterosexual individuals.	radio (Matrix), Required<br>1 Strongly disagree1<br>2 2<br>3 Neither agree nor disagree3<br>4 4<br>5 Strongly agree5
	78	[docss_lgbdiscreet]<br>Show the field ONLY if:<br>[age] >= 18	LGB individuals must be discreet about their sexual orientation around children.	radio (Matrix), Required<br>1 Strongly disagree1<br>2 2<br>3 Neither agree nor disagree3<br>4 4<br>5 Strongly agree5
	79	[docss_transmoreprob]<br>Show the field ONLY if:<br>[age] >= 18	I am aware of research indicating that transgender individuals experience disproportionate levels of health and mental health problems compared to cisgender individuals.	radio (Matrix), Required<br>1 Strongly disagree1<br>2 2<br>3 Neither agree nor disagree3<br>4 4<br>5 Strongly agree5
	80	[docss_transdeviant]<br>Show the field ONLY if:<br>[age] >= 18	When it comes to transgender individuals, I believe they are morally deviant.	radio (Matrix), Required<br>1 Strongly disagree1<br>2 2<br>3 Neither agree nor disagree3<br>4 4<br>5 Strongly agree5
	81	[docss_transtrain]<br>Show the field ONLY if:<br>[age] >= 18	I have received adequate clinical training and supervision to work with transgender clients/patients.	radio (Matrix), Required<br>1 Strongly disagree1<br>2 2<br>3 Neither agree nor disagree3<br>4 4<br>5 Strongly agree5
	82	[docss_lgbtrain]<br>Show the field ONLY if:<br>[age] >= 18	I have received adequate clinical training and supervision to work with lesbian, gay, and bisexual (LGB) clients/patients.	radio (Matrix), Required<br>1 Strongly disagree1<br>2 2<br>3 Neither agree nor disagree3<br>4 4

				5 Strongly agree5
	83	[docss_lgbimmoral]<br>Show the field ONLY if:<br>[age] >= 18	The lifestyle of a LGB individual is unnatural or immoral.	radio (Matrix), Required<br>1 Strongly disagree1<br>2 2<br>3 Neither agree nor disagree3<br>4 4<br>5 Strongly agree5
	84	[docss_lgbexp]<br>Show the field ONLY if:<br>[age] >= 18	I have experience working with LGB clients/patients.	radio (Matrix), Required<br>1 Strongly disagree1<br>2 2<br>3 Neither agree nor disagree3<br>4 4<br>5 Strongly agree5
	85	[docss_lgbcomp]<br>Show the field ONLY if:<br>[age] >= 18	I feel competent to assess a person who is LGB in a therapeutic setting.	radio (Matrix), Required<br>1 Strongly disagree1<br>2 2<br>3 Neither agree nor disagree3<br>4 4<br>5 Strongly agree5
	86	[docss_transassess]<br>Show the field ONLY if:<br>[age] >= 18	I feel competent to assess a person who is transgender in a therapeutic setting.	radio (Matrix), Required<br>1 Strongly disagree1<br>2 2<br>3 Neither agree nor disagree3<br>4 4<br>5 Strongly agree5
	87	[docss_transexp]<br>Show the field ONLY if:<br>[age] >= 18	I have experience working with transgender clients/patients.	radio (Matrix), Required<br>1 Strongly disagree1<br>2 2<br>3 Neither agree nor disagree3<br>4 4<br>5 Strongly agree5
	88	[docss_dressperv]<br>Show the field ONLY if:<br>[age] >= 18	People who dress opposite to their biological sex have a perversion.	radio (Matrix), Required<br>1 Strongly disagree1<br>2 2<br>3 Neither agree nor disagree3<br>4 4<br>5 Strongly agree5

	89	[docss_uncomfort]<br><br>Show the field ONLY if:<br>[age] >= 18	I would be morally uncomfortable working with a LGBT client/patient.	radio (Matrix), Required<br>1 Strongly disagree1<br>2 2<br>3 Neither agree nor disagree3<br>4 4<br>5 Strongly agree5
	90	[clim_respect]<br><br>Show the field ONLY if:<br>[age] >= 18	Section Header: *LGBT Climate InventoryInstructions: Please rate the following items according to how well they describe the atmosphere for lesbian, gay, bisexual, and transgendered (LGBT) employees in your workplace (or placement if you are a student), using the following scale where 1= Doesn't describe at all to 4= Describes Extremely Well.*<br><br>Lesbian, gay, bisexual, and transgender (LGBT) employees are treated with respect.	radio (Matrix), Required<br>1 Doesn't describe at all1<br>2 Describes Somewhat or a Little2<br>3 Describes Pretty Well3<br>4 Describes Extremely Well4
	91	[clim_question]<br><br>Show the field ONLY if:<br>[age] >= 18	Coworkers are as likely to ask nice, interested questions about a same-sex relationship as they are about a heterosexual relationship.	radio (Matrix), Required<br>1 Doesn't describe at all1<br>2 Describes Somewhat or a Little2<br>3 Describes Pretty Well3<br>4 Describes Extremely Well4
	92	[clim_comfort]<br><br>Show the field ONLY if:<br>[age] >= 18	LGBT people consider it a comfortable place to work.	radio (Matrix), Required<br>1 Doesn't describe at all1<br>2 Describes Somewhat or a Little2<br>3 Describes Pretty Well3<br>4 Describes Extremely Well4
	93	[clim_humor]<br><br>Show the field ONLY if:<br>[age] >= 18	Non-LGBT employees are comfortable engaging in gay-friendly humour with LGBT employees (e.g., joking about a date).	radio (Matrix), Required<br>1 Doesn't describe at all1<br>2 Describes Somewhat or a Little2<br>3 Describes Pretty Well3<br>4 Describes Extremely Well4
	94	[clim_accept]<br><br>Show the field ONLY if:<br>[age] >= 18	LGBT employees feel accepted by coworkers.	radio (Matrix), Required<br>1 Doesn't describe at all1<br>2 Describes Somewhat or a Little2<br>3 Describes Pretty Well3<br>4 Describes Extremely Well4
	95	[clim_support]<br><br>Show the field ONLY if:<br>[age] >= 18	My immediate work group is supportive of LGBT coworkers.	radio (Matrix), Required<br>1 Doesn't describe at all1<br>2 Describes Somewhat or a Little2<br>3 Describes Pretty Well3<br>4 Describes Extremely Well4

	96	[clim_talk]<br><br>Show the field ONLY if:<br>[age] >= 18	LGBT employees are comfortable talking about their personal lives with coworkers.	radio (Matrix), Required<br>1 Doesn't describe at all1<br>2 Describes Somewhat or a Little2<br>3 Describes Pretty Well3<br>4 Describes Extremely Well4
	97	[clim_noissue]<br><br>Show the field ONLY if:<br>[age] >= 18	Employee LGBT identity does not seem to be an issue.	radio (Matrix), Required<br>1 Doesn't describe at all1<br>2 Describes Somewhat or a Little2<br>3 Describes Pretty Well3<br>4 Describes Extremely Well4
	98	[clim_environment]<br><br>Show the field ONLY if:<br>[age] >= 18	The company or institution as a whole provides a supportive environment for LGBT people.	radio (Matrix), Required<br>1 Doesn't describe at all1<br>2 Describes Somewhat or a Little2<br>3 Describes Pretty Well3<br>4 Describes Extremely Well4
	99	[clim_free]<br><br>Show the field ONLY if:<br>[age] >= 18	LGBT employees are free to be themselves.	radio (Matrix), Required<br>1 Doesn't describe at all1<br>2 Describes Somewhat or a Little2<br>3 Describes Pretty Well3<br>4 Describes Extremely Well4
	100	[clim_display]<br><br>Show the field ONLY if:<br>[age] >= 18	LGBT employees feel free to display pictures of same-sex partners.	radio (Matrix), Required<br>1 Doesn't describe at all1<br>2 Describes Somewhat or a Little2<br>3 Describes Pretty Well3<br>4 Describes Extremely Well4
	101	[clim_improve]<br><br>Show the field ONLY if:<br>[age] >= 18	The atmosphere for LGBT employees is improving.	radio (Matrix), Required<br>1 Doesn't describe at all1<br>2 Describes Somewhat or a Little2<br>3 Describes Pretty Well3<br>4 Describes Extremely Well4
	102	[concernsuggest]<br><br>Show the field ONLY if:<br>[age] >= 18	Section Header: *And finally...*<br>Do you have any suggestions or comments?	notes<br>Custom alignment: LV
	103	[progress_3]	Survey 100% Complete	descriptive
	104	[pride_scale_uk_version_complete]	Section Header: *Form Status*<br>Complete?	dropdown<br>0 Incomplete<br>1 Unverified<br>2 Complete
